# A Rare Case of Syndrome of Inappropriate Anti-diuretic Hormone in Non-small Cell Lung Cancer Presenting as Superior Vena Cava Syndrome

**DOI:** 10.7759/cureus.4861

**Published:** 2019-06-07

**Authors:** Rhys Ishihara, Catherine Stoos, Sunil Jagadesh, Cam Nguyen

**Affiliations:** 1 Radation Oncology, Creighton University School of Medicine, Omaha, USA; 2 Pathology, Creighton University School of Medicine, Omaha, USA; 3 Nephrology, Creighton University School of Medicine, Omaha, USA; 4 Radiation Oncology, Creighton University School of Medicine, Omaha, USA

**Keywords:** svc syndrome, siadh, lung adeno, non-small cell lung cancer, non-small cell lung cancer

## Abstract

Syndrome of inappropriate anti-diuretic hormone (SIADH) secretion is uncommon in small cell lung cancer (SCLC), but even more rare in cases of non-small cell lung cancer (NSCLC). We report a case of a 59-year-old male who presented with superior vena cava (SVC) syndrome. After further investigation, he was diagnosed with adenocarcinoma of the lung. He delayed his medical care and his condition worsened. He was diagnosed with SIADH as an incidental finding on routine lab draw. Radiotherapy was subsequently initiated, and after one week of treatment, the patient showed marked clinical improvement. In this article, we also review the current indications for radiotherapy in various lung cancers and the management of SIADH.

## Introduction

Syndrome of inappropriate anti-diuretic hormone (SIADH) secretion occurs when there is increased secretion of anti-diuretic hormone (ADH). There are many reported causes of SIADH which include, drugs, central nervous system (CNS) disturbances, malignancy, surgery, and human immunodeficiency virus (HIV) infection. SIADH typically presents as hyponatremia with an inappropriately elevated urine osmolality in a euvolemic patient [1]. The prevalence of SIADH in small cell lung cancer (SCLC) is estimated to be from 7%-16% [2]. However, it is estimated that 70% of all SIADH due to malignancy is attributable to SCLC [2]. Other malignancies that can cause SIADH include pancreatic, duodenal, head and neck cancers [1]. Although the prevalence of SIADH in non-small cell lung cancer (NSCLC) is unknown, there have been only four cases reported in the past two decades [3-6]. 

## Case presentation

**Figure 1:**
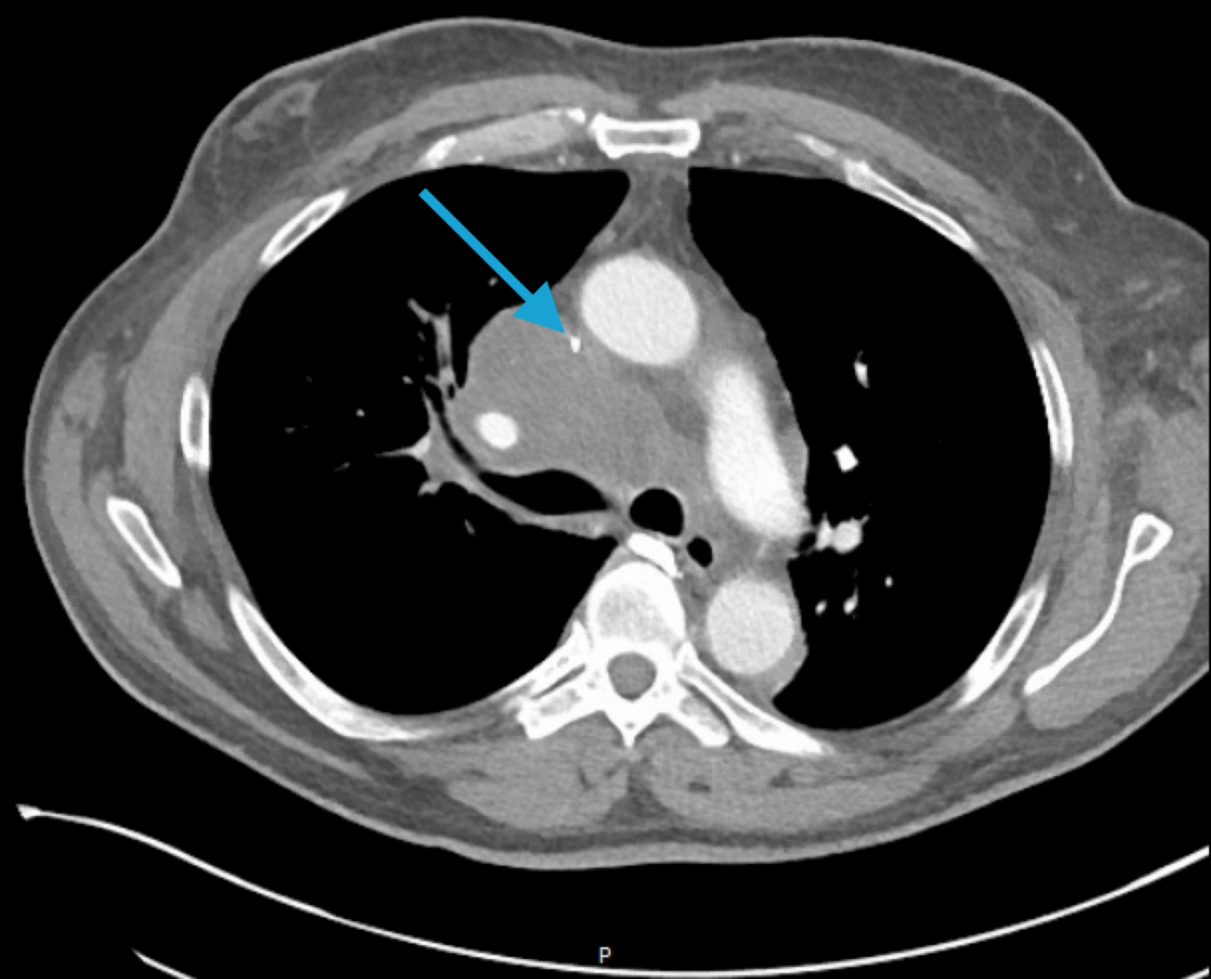
Computed tomography (CT) scan of the chest showing the right paratracheal mass encasing the superior vena cava (SVC) (indicated by blue arrow).

**Figure 2:**
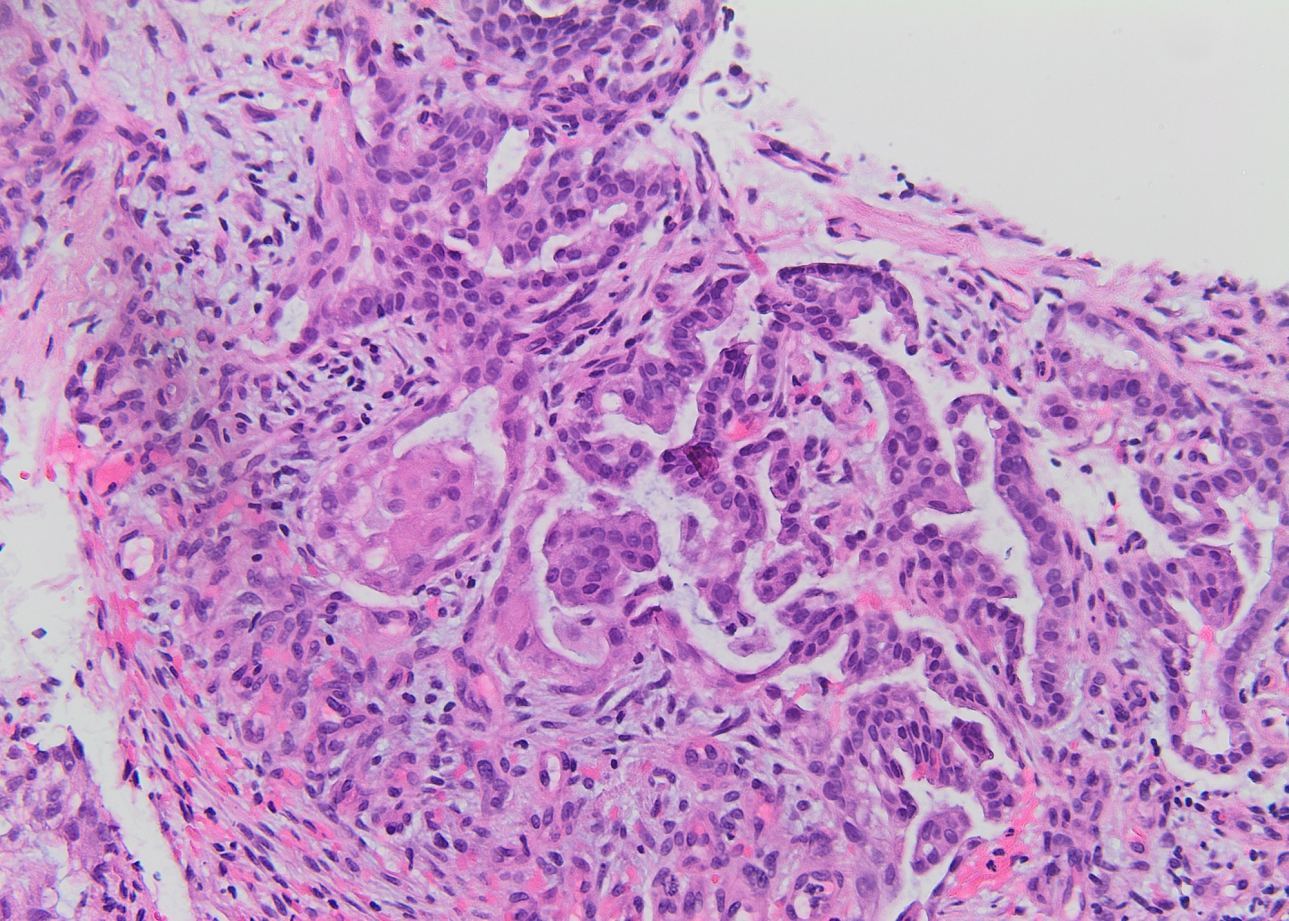
Examination of the lung biopsy. The lung biopsy revealed a tumor composed of malignant cells forming glands, consistent with well to moderately differentiated adenocarcinoma. The tumor was present within a desmoplastic background. Immunohistochemical stains were performed, and the results were in keeping with adenocarcinoma of lung origin. In some areas, the tumor takes on a vaguely papillary architecture.

**Figure 3:**
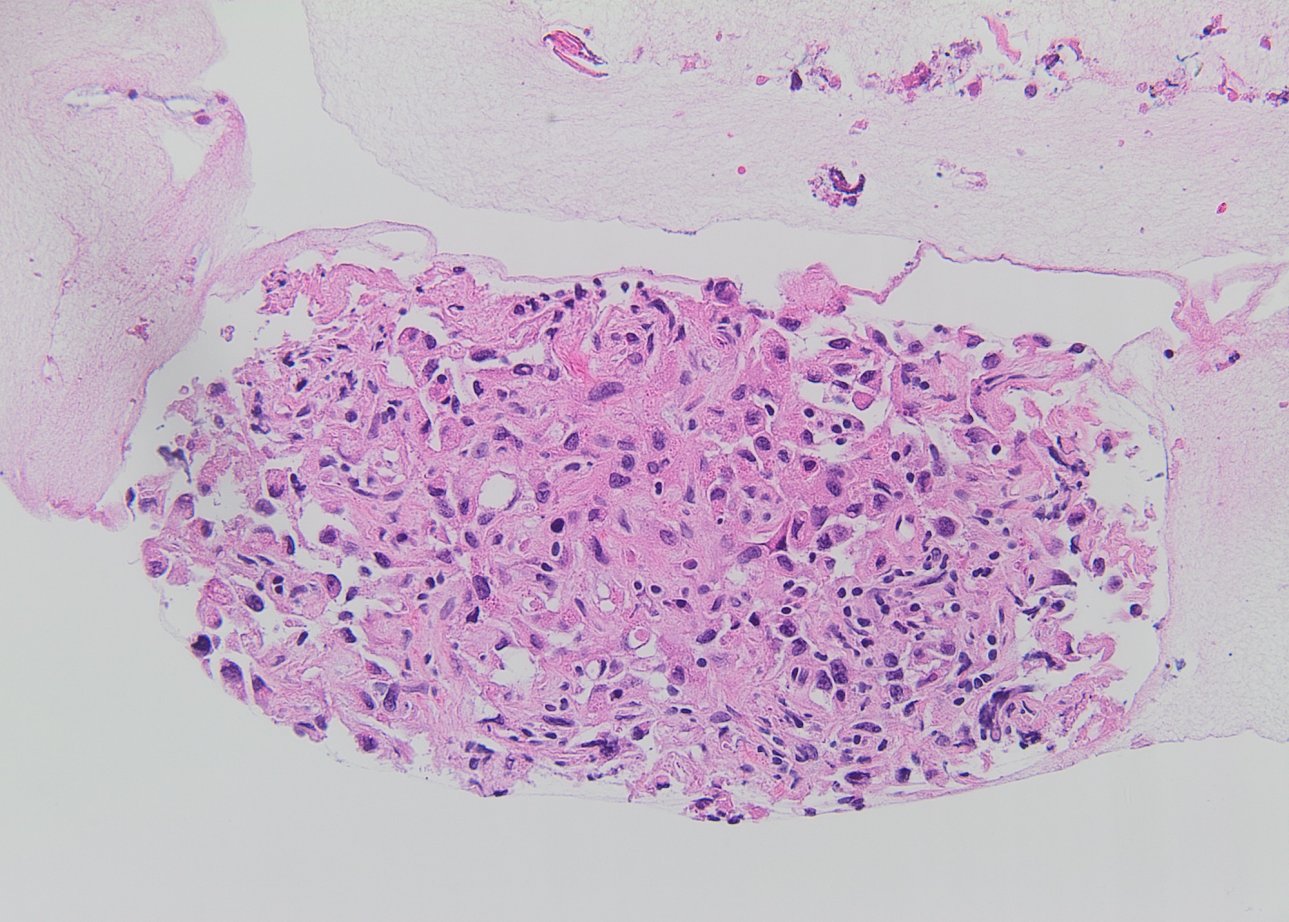
Several lymph nodes demonstrated poorly-differentiated, metastatic, non-small cell carcinoma, composed of tumor cells with high nuclear pleomorphism within a fibrotic background. Tumor necrosis was also present.

A 59-year-old male with a past medical history of coronary artery disease, left ventricular aneurysm, atrial fibrillation, stroke and left below knee amputation due to gangrene, presented to the emergency department (ED) with moderate facial swelling for three days. The swelling began in his neck and became more prominent around his eyes and lips. He is a current smoker with a 47-pack-year smoking history. His family history was unremarkable. He is currently taking apixaban, atorvastatin, and metoprolol. Physical exam showed periorbital, facial, and perioral edema. The patient received dexamethasone and diphenhydramine in the ED with no clinical improvement. A computed tomography (CT) scan of his neck and chest showed a right paratracheal mass measuring 5 x 3.8 cm encasing the right pulmonary artery and narrowing of the superior vena cava (SVC) (Figure 1). In addition, the CT scan showed lytic lesions in the spine and a left adrenal mass suspicious for metastatic disease. Tissue biopsy via endobronchial ultrasound showed adenocarcinoma of the lung (Figures 2-3). Furthermore, the patient’s facial swelling was attributed to SVC syndrome. The patient left the hospital against medical advice before receiving any treatment for his disease.

The patient returned to the ED five days later with more prominent facial swelling and dyspnea. His vital signs were within normal limits and physical exam was notable for worsening of his facial edema and excessive hoarseness of his voice. The patient began radiotherapy of the NSCLC 3000 cGy for 10 fractions. On routine basic metabolic panel, his sodium was 122 mg/dL (135-145 mg/dL) and measured serum osmolality was 264 mOsm/kg (275-295 mOsm/kg). His urine osmolality and sodium were inappropriately normal at 786 mOsm/kg (500-800 mOsm/kg) and 23 mmol/L (20-110 mmol/L), respectively. Other common causes of SIADH were ruled out (medications or brain injury) and he was diagnosed with SIADH due to NSCLC. His sodium was corrected slowly through hypertonic saline (3%) and 2 g salt tablets. Seven days after beginning treatment with radiation, his SVC syndrome markedly improved and his sodium was within normal limits.

## Discussion

Lung cancer is the second most common malignancy in the United States and is the leading cause of death from cancer in both men and women. The American Cancer Society estimates 228,150 new cases of lung cancer and 142,670 deaths from lung cancer in 2019 [6]. Lung cancer is split into two different major subtypes, SCLC and NSCLC. NSCLC is further subdivided into adenocarcinoma, squamous cell carcinoma and large cell carcinoma, and comprises most of all lung cancers, about 85% [7]. Typically, patients present with nonspecific symptoms of cough, hemoptysis, chest pain, and dyspnea. In addition, they will be positive for risk factors, the most important being cigarette smoke [8]. Like in our patient, adenocarcinoma of the lung is diagnosed by immunohistochemistry to stain positive for thyroid transcription factor 1 (TTF-1) and/or Napsin A [9].

SVC syndrome is usually a radiation oncologic emergency that requires quick diagnosis and treatment. SVC syndrome is most commonly caused by malignancy, the majority being lung cancer (65%) and the minority being lymphoma (35%) [10]. SVC syndrome is a clinical diagnosis and the most common presenting symptoms are dyspnea and facial edema. Biopsy and histologic diagnosis should be obtained before proceeding with treatment. Depending on histologic classification of the tumor, treatment will vary. In a curative situation where NSCLC is confined to the lung, chemotherapy and radiotherapy can be given using carboplatin and paclitaxel [11] with 6000 cGy in 30 fractions. However, most cases of NSCLC with SVC syndrome show very advanced tumor progression with distant metastases. In these cases, radiotherapy alone is given for 3000 cGy in 10 fractions for palliative purposes. On the other hand, cases of SCLC and lymphoma are typically treated with chemotherapy alone with consolidative radiotherapy and for treatment of bulky disease in each of the cancers, respectively [10].

In rare cases, SVC syndrome can be caused by benign etiologies such as Port-a-Cath, fibrosing mediastinitis, tuberculosis, retrosternal goiters, or radiation fibrosis to name a few. Treatment for these cases usually consists of antibiotics or anticoagulation depending on what is indicated for the cause of the underlying disorder [12].

Paraneoplastic syndromes occur as a result of tumors that secrete hormones or other factors that can cause symptoms unrelated to the malignancy. Paraneoplastic syndromes that are associated with lung malignancies include hypercalcemia of malignancy and SIADH. The incidence of paraneoplastic syndromes in lung cancer is low overall and occurs in around 10% of all cases. NSCLC is more likely to be associated with hypercalcemia of malignancy than SCLC, but it can occur in both subtypes [13]. However, SIADH, if present, is usually associated with SCLC and very few cases have been reported with NSCLC. About 70% of all cases of SIADH caused by a paraneoplastic syndrome are associated with SCLC, but SIADH only occurs in 7%-16% of all SCLC cases. Additionally, in these cases, SIADH is a poor prognostic factor is indicative of inferior survival outcomes for patients [14].

The SIADH is a disorder of impaired water excretion caused by the inability to suppress the secretion of antidiuretic hormone. Besides malignancy, additional causes of SIADH include CNS abnormalities (trauma, stroke or hemorrhage), drugs (carbamazepine, oxcarbazepine, chlorpropamide, cyclophosphamide, and selective serotonin reuptake inhibitors (SSRIs)) and other lung diseases [1].

The signs and symptoms of SIADH are dependent on the degree of hyponatremia and the rapidity of the onset of hyponatremia. Patients with severe hyponatremia (defined as sodium less than 120 mEq/L) will have symptoms which include headache, weakness, seizures and altered mental status. These symptoms will generally occur at a sodium decreases to less than 120 mEq/L over 48 hours or less. Diagnosis of SIADH is made using the Bartter and Schwartz criteria which include decreased plasma osmolality (< 275 mOsm/kg), inappropriately concentrated urine (> 100 mOsm/kg) with increased urine concentration of sodium (> 40 mEq/L), euvolemia, and normal thyroid, adrenal and renal function [1].

Effective treatment of SIADH involves treating the underlying cause; in our case, it was radiotherapy to the tumor. However, correction of sodium is commonly performed. This is achieved through oral salt tablets, hypertonic (3%) saline and fluid restriction. Rapid correction of sodium in a hyponatremic patient may result in osmotic demyelination syndrome which is characterized by irreversible altered mental status, paraparesis or quadriparesis, movement disorders, seizures, lethargy and coma [15]. Therefore, the current recommendation is that the rate of sodium correction should not exceed 6-8 mEq/L in the first 24 hours and 16 mEq/L in the first 48 hours [16]. Pharmacotherapy can also be initiated. Urea can be given in a dose of 30 g/day to facilitate water excretion through the urine but rarely used. ADH receptor antagonists, like tolvaptan, satavaptan and conivaptan can be used, but there is an increased risk of rapid correction of sodium. Demeclocycline, a tetracycline derivative, may also be used but is potentially nephrotoxic [1].

Of the four cases reported in the past, two of the patients were diagnosed with squamous cell carcinoma and two were diagnosed with adenocarcinoma. McDonald et al. reports presentation of their patient's SIADH coinciding with the initiation of radiation therapy [5]. Both Katsuragi et al. and Tho et al. report improvement of SIADH with treatment with lung resection and MIC chemotherapy (mitomycin, ifosfamide, and cisplatin), respectively [3-4]. Lastly, Iyer et al. treated the SIADH with salt supplementation, but did not treat the patient with radiation or chemotherapy due to brain metastasis [6]. This patient's hyponatremia improved, but still remained below the normal limits. Our case is unique in that the patient's SIADH improved shortly after treatment with radiation therapy. Clinically, the patients SIADH improved concurrently with improvement of SVC syndrome. This is the first report of improvement of SIADH in NSCLC with radiation therapy without chemotherapy.

## Conclusions

In our case, our patient presented with initial SVC syndrome and was found to have a lung mass obstructing his SVC. The diagnosis of lung adenocarcinoma was confirmed via tissue biopsy. On readmission after leaving against medical advice, he was found to be hyponatremic with an elevated urine osmolarity. This confirmed the diagnosis of SIADH in our patient. SIADH is an extremely rare paraneoplastic syndrome when it is associated with NSCLC. To the best of our knowledge, only four cases have been published about this association within the last two decades. In addition, our patient showed clinical improvement of SIADH after receiving radiation therapy. This case illustrates that even patients with NSCLC are at risk for SIADH as a paraneoplastic syndrome. Clinicians should be aware that SIADH is a possible sequalae of both major subtypes of primary lung malignancy.
